# Diagnosis of Lesions of the Heart Previous to Administering Anæsthetics

**Published:** 1896-01

**Authors:** W. H. Whitslar


### Diagnosis of Lesions of the Heart Previous to Administering Anæsthetics.

BY DR. W. H. WHITSLAR, D.D.S.

Read before the Ohio State Dental Society, December 4, 1895.

It is a serious problem when anæsthetics are given, to whom and under what conditions they may be administered. This is of such importance that every practitioner should consider well the advisability of assuming the responsibility of the care of an individual in an unconscious state. The topic before us, therefore, opens up to my mind these questions:

(*a*) Do lesions of the heart interfere with anæsthesia?
(*b*) Is diagnosis of the lesions an easy matter?
(*c*) How may diagnosis be made?
(*d*) What are the responsibilities?

Let us consider these questions. (*a*) Every human body has three circulating fluids, i.e., blood, lymph and chyle. The blood in man is proportioned to the weight of the body, as one is to thirteen, and this ratio is indicative of physical value aside from that of the vital. Of course we realize its worth in vital organization, because it is the provider of foods to the various tissues. It is also the scavenger that takes away impurities. It at one time or another contains everything about to become part of the tissues and everything which has ceased to belong to them. Morphologically, blood consists of plasm and corpuscles, red and white, also blood platelets. Normally, there exists in one cubic millimeter of plasm five millions of red corpuscles and three hundred thousand blood platelets. There is one white-blood corpuscle (leucocyte) for every five hundred red corpuscles. Blood being a circulating fluid, it is attended by three factors, i.e., its propelling force, fluidity, and size of vessels. All these, the heart, the plasm, thickened or thinned by numbers of corpuscles, and the lumen and area of vessel walls, modify the velocity of the blood current. Disturbances in either of these factors produce changes in the circulation of the blood. Now, if the circulation of the blood be disturbed by anything for an undue length of time, disease is the result. We may, however, cause a transgression from the natural course for a short time by emotion, change of position of the body (gravitation), over-indulgence in food, and not seriously affect the blood currents, but these would, if too pronounced, manifest unusual disturbances in the action of the heart, therefore, from so simple an illustration it is easy to conceive changes wrought in the blood by the introduction of a vapor that poisons its composition sufficiently to produce that state known as anæsthesia. These changes are rendered less agreeable to the vital economy in proportion to the diseased conditions. Hence, lesions affect anæsthesia proportionately to their diseased condition and less proportionately to their compensated states found with lesions of the heart, simply because of the vitality of the organs being insufficient to resist greater ingress of unnatural bodies. The blood itself has most to do with anæsthesia, but this cannot move continuously without a propelling force, and if that be weakened you perceive the result.

(*b and c*) The greatest modifications of the circulating apparatus are to be found in the heart which forces blood to every nook and cranny of the body. The problem of knowledge of the diseases that affect the heart's action is partly physical, partly vital. Diseases of the circulatory apparatus are associated to a great extent with certain mechanical disturbances called lesions. This is owing to the fact that these organs subserve mechanical purposes and the regular rhythm is maintained by the pump arrangement and its distributory canals. To understand this class of diseases one must resort to physical methods of examination. Three of the great senses are used in this examination which includes an *Inspection* of the size and form of body externally; *Palpitation*, feeling the movements of walls of the thorax as well as heart; *Percussion*, which relates the condition of density by sounds; and *Auscultation*, also relating to sounds more specific in character. The pulse plays a significant part, as I will illustrate later on.

In order to comprehend the various lesions which produce sounds, or other defects, one must be familiar with the normal heart's action. Normally, there are six sounds of the heart, as follows: two vibrations of the auricular-ventricular valves, two vibrations of semi-lunar valves, and two vibrations of walls of large vessels. Generally speaking these are resolved into two sounds, the systolic or first sound, by the contraction of the ventricles felt at the apex of the heart, and the diastolic, or second sound, heard at the base of the heart, by the sigmoid valves of the aorta and pulmonary artery.

All these sounds are functional; if altered they become organic, and then it is that we perceive disease. Alterations may arise from fevers, fatty degenerations or hypertrophies. Want of synchronous action of the valves of the two sides split one or the other into component elements so that either sound may be re-duplicated. Any condition which increases the lesion, either in the systemic or pulmonary circulation, may disturb the balance of the two sides so as to dispose to a re-duplication of the sounds.

Cardiac sounds may be replaced by murmurs in the heart or outside of it. These have a blowing, rasping, sighing, and other characteristics. To determine these and their significance, two important facts must be studied, namely their rhythm and their area of distribution.

The diseases of the heart and its valves which produce mechanical disorders of the circulation by establishing abnormal relations, are of two kinds: obstructive and regurgitant.

Valvular disease, narrowing the size of the orifice, presents an obstacle to the passage of the blood currents, a condition better known as *stenosis*. On the other hand, when the blood flows back through an orifice of imperfect closure of the valves, due either to widening of the orifice or valvular changes, the condition is called regurgitation or insufficiency. Each of the orifices may be affected with one or both forms of the disease, but the frequency with which the several orifices are attacked varies. Generally the left side of the heart is affected by organic disease. The right is disturbed by lung diseases which cause increased tension leading to dilatation. All valvular diseases tend to lessen the arterial blood and there is an over-fulness and a stasis of the veins. From this there follows visceral disorders.

This is often diverted by compensation to form a normal balance. In this we find that hyphertrophy assists by increasing the contractile power behind the defective valve. This produces greater tension in the vessels. Any failure of compensation is due to loss of nutrition, then occurs palpitation from slight exertion or excitement. From thirty to forty per cent. of all cases unassociated with other lesions are of *mitral* insufficiency. (Pepper.) Under chloroform, heart failure as a rule is due to fatty degeneration or mitral or aortic valvular lesions.

Now to diagnose these various lesions, endocarditis, myocarditis may be present and affect the tissues so as to produce alterations of sounds or conditions. Then, too, temporary affections of the heart may be produced by diseases of the liver. Murchison states that it is not rare to observe enfeebled circulation in hepatic disorder. It is believed by Faber and others that biliary acids have direct action upon the cardiac muscle. Independently of palpitations, syncope, and painful affections, nervous disorders, cephalalgia, insomnia, hallucinations, and so on, are secondary to hyphertrophy as a sign of disease. A common sign is palpitation. This is sometimes symptomatic of dyspepsia, therefore is not pathognomonic. It is on this account that many persons think they have heart disease, when it is only a reflex irritation from the digestive organs. It is often supposed by the ordinary observer that intermittency of the pulse is an indication of heart lesions. This is common in men of advanced age. It is a nervous trick and bears no significance if it is a pure halt of the heart. Intermittency with irregularity may be found in fatty degeneration of the heart or during fevers. In valvular disease the value of the pulse is best known by the use of the smygmograph, but this is generally impracticable because it does not assist as much as the physical diagnosis directly over the heart by the methods already mentioned. Now, I mention these facts to show that diagnosis of lesions of the heart is not an easy matter, and that a knowledge of not only the heart but lungs and visceral organs is required to comprehend fully cardiac diseases. By the fact of difficulty of diagnosis of these affections it is more apparent that responsibility is serious. The use of an anæsthetic is attended with appreciable risk and the life of the patient is endangered. Occasionally, there is a death. Dr. William Pepper states, that "on the whole, sudden death is rare from valvular disease." This favors, if true, the ignorant man who uses an anæsthetic and there is no doubt it saves many lives that are jeopardized by ignorance. The law, however, does not recognize ignorance, even if the ignorant be careful. He must have a knowledge, then skill.

